# Enhancement of antireflection property of silicon using nanostructured surface combined with a polymer deposition

**DOI:** 10.1186/1556-276X-9-9

**Published:** 2014-01-08

**Authors:** Jun Mok Ha, Sung Ho Yoo, Jong Hoi Cho, Yong Hoon Cho, Sung Oh Cho

**Affiliations:** 1 Department of Nuclear and Quantum Engineering, Korea Advanced Institute of Science and Technology, 373-1 Guseong, Yuseong, Daejeon 305-701, Republic of Korea; 2 Department of Physics and KI for the NanoCentury, Korea Advanced Institute of Science and Technology, 373-1 Guseong, Yuseong, Daejeon 305-701, Republic of Korea

**Keywords:** Silicon nanostructure, Antireflection, Hydrogen etching, Silicon-based polymer

## Abstract

Silicon (Si) nanostructures that exhibit a significantly low reflectance in ultraviolet (UV) and visible light wavelength regions are fabricated using a hydrogen etching process. The fabricated Si nanostructures have aperiodic subwavelength structures with pyramid-like morphologies. The detailed morphologies of the nanostructures can be controlled by changing the etching condition. The nanostructured Si exhibited much more reduced reflectance than a flat Si surface: an average reflectance of the nanostructured Si was approximately 6.8% in visible light region and a slight high reflectance of approximately 17% in UV region. The reflectance was further reduced in both UV and visible light region through the deposition of a poly(dimethylsiloxane) layer with a rough surface on the Si nanostructure: the reflectance can be decreased down to 2.5%. The enhancement of the antireflection properties was analyzed with a finite difference time domain simulation method.

## Background

Recently, antireflection (AR) techniques have been widely used in various applications such as solar cells [1-3], electro-optical devices [4], sensors [5], and lenses [6] to significantly suppress the reflective loss at the interface of two media. In particular, in solar cells using crystalline silicon (Si) modules, AR has been a significant research focus due to the enhancement of photo-conversion efficiency [1,2]. Despite excellent conversion efficiency in crystalline Si solar cells, the high refractive index (*n* = 3.4) of Si has limited the efficient utilization of sunlight [7,8]. This is because more than 30% of incident sunlight is scattered or reflected from the Si surface due to a large discontinuity of *n* between the air and Si interface.

In order to reduce the reflection from the air-material interface, the *n* of the two media should be similar or changed smoothly at the interface. Nature has its own strategy to effectively reduce reflection: for example, nanostructured surface on a moth eye [6,9]. Such biological nanostructured surfaces can create a composite comprising air and a material, where *n* gradually changes from the air to the material because effective *n* depends on the volume fraction of the two media. Furthermore, it is important to note that moth eyes are satisfied that they have the optimal AR conditions using two-dimensional subwavelength structures [4,10] and tapered morphologies [4,11].

So far, several types of biomimetic nanostructured surfaces with excellent AR properties have been developed using electron-beam lithography, laser interference lithography, and nanoimprint lithography [12-14]. However, these techniques require expensive devices and complicated procedures. Moreover, there have been few papers that describe simple post-treatments to further reduce the reflection from the material surface, although some post-treatment methods have been reported including oxygen treatments for improving the abrasion resistance of the coating [15], NH_3_-heat processes followed by a trimethylchlorosilane modification to enhance the scratch resistance and moisture resistance [16], and the effects of heat, laser, and ion post-treatments on HfO_2_ single layers [17]. Here, we present a hydrogen etching approach to fabricate pyramid-shaped Si nanostructures that exhibits a comparatively low reflectance at the wavelength regions of ultraviolet (UV) and visible (Vis). The aspect ratio and two-dimensional spacing of Si nanostructures can be controlled by changing the etching condition. In addition, the reflectance was further reduced by depositing a Si-based polymer on the fabricated Si nanostructures, which also induce more uniform reflectance behavior over UV and Vis regions.

## Methods

**Figure 1:**
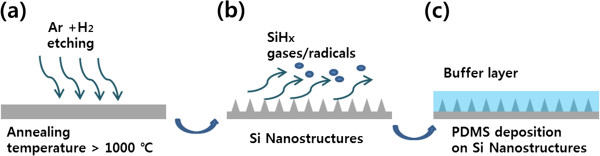
**Schematics of the fabrication process for the Si nanostructures. (a, b)** The Si sheets were etched using hydrogen and argon mixture gases under 1 × 10^−2^ Torr at different high temperatures. **(c)** The Si-based polymer (PDMS) deposition on the Si nanostructures for enhancing the AR property.

The fabrication process of the Si nanostructures is displayed schematically in Figure 1. A polished (100) Si plate (10 × 10 mm^2^) (p-type; Namkang Hi-Tech Co., Sungnam, South Korea) was washed by isopropyl alcohol (Sigma Aldrich, St. Louis, MO, USA) and dried using nitrogen gas in order to remove impurities on the Si plate. After cleaning the Si plate, the hydrogen etching process was conducted using hydrogen (10%) and argon (90%) mixture gases under 1 × 10^−2^ Torr at different temperatures (1,350°C, 1,200°C, and 1,100°C). The holding time at the maximum annealing temperature was 30 min and the flow rate of mixture gases was 0.5 standard cubic centimeters per minute (sccm) during the annealing process. Subsequently, a poly(dimethylsiloxane) (PDMS) (viscosity 2,000,000 cSt) (Dow Corning, Jincheon, Chungbuk, South Korea) layer was deposited on the fabricated Si nanostructures through a doctor blade technique [18] to enhance the AR property. The thickness of the PDMS layer was approximately 1 μm. The morphologies of the fabricated Si nanostructures were characterized using a field emission scanning electron microscope (FESEM; Hitachi S-4800, Hitachi, Tokyo, Japan). The roughness of the PDMS surface on the Si nanostructures was measured using an atomic force microscope (AFM; XE-70, Park Systems, Ft. Lauderdale, FL, USA). The AR properties of the Si nanostructures were analyzed using a finite difference time domain (FDTD) simulation method and measured using the diffuse reflectance (DR) module of an UV–Vis spectrometer (SCINCO S-4100, SCINCO, Daejeon, South Korea). A xenon (Xe) lamp was used as the light source at wavelengths of 300 to 800 nm. The measurement error of the UV–Vis spectrometer was less than 0.1% by subtracting the unstable wavelength regions of the Xe lamp (190 to 200 nm and 900 to 1,100 nm) in advance.

## Results and discussion

**Figure 2:**
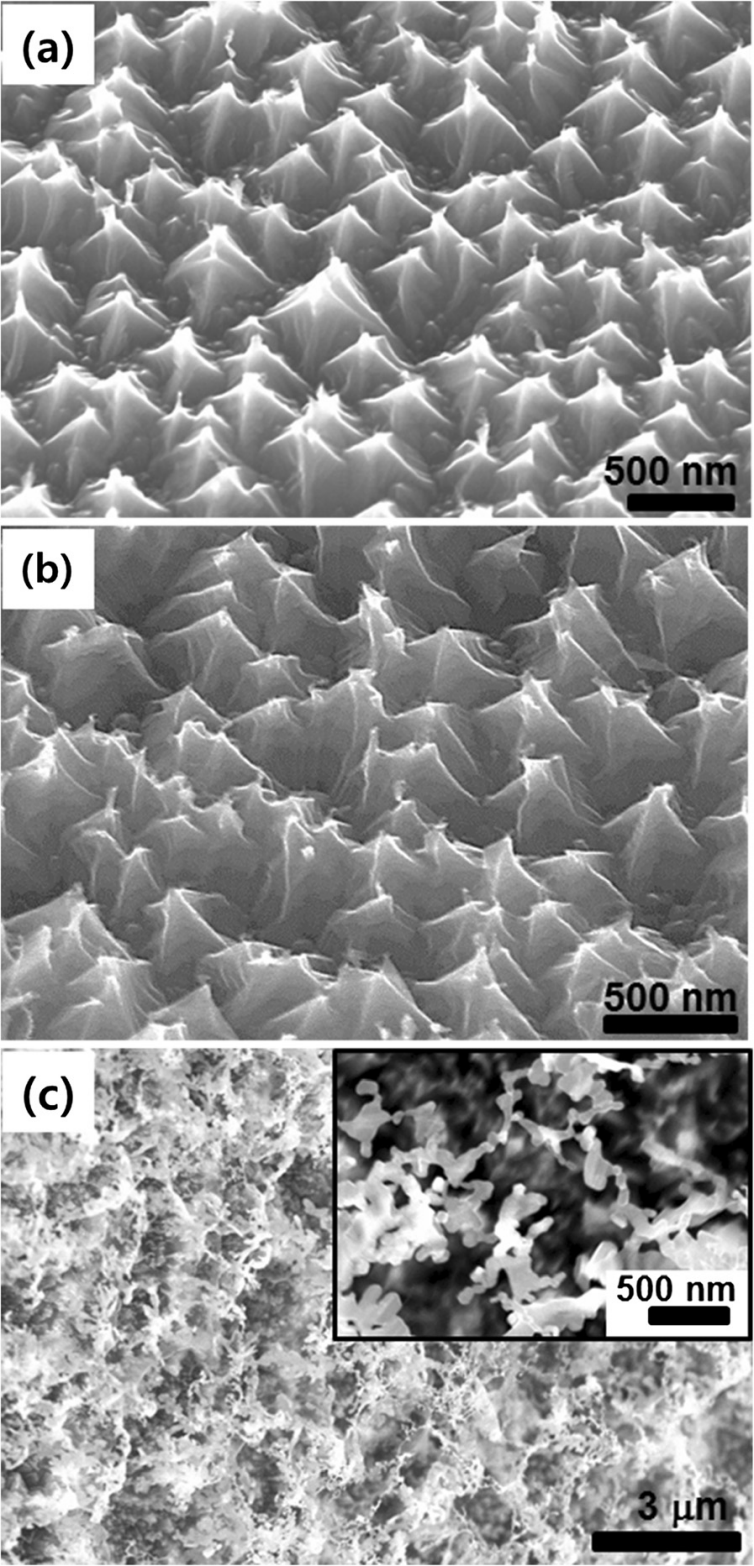
**Tilted FESEM images of the Si nanostructures etched by various flow rates of mixture gas. (a)** 0.5 sccm. **(b)** 2.5 sccm. **(c)** 5.0 sccm. Inset: magnified FESEM images of the aggregate of nanoparticles.

**Figure 3:**
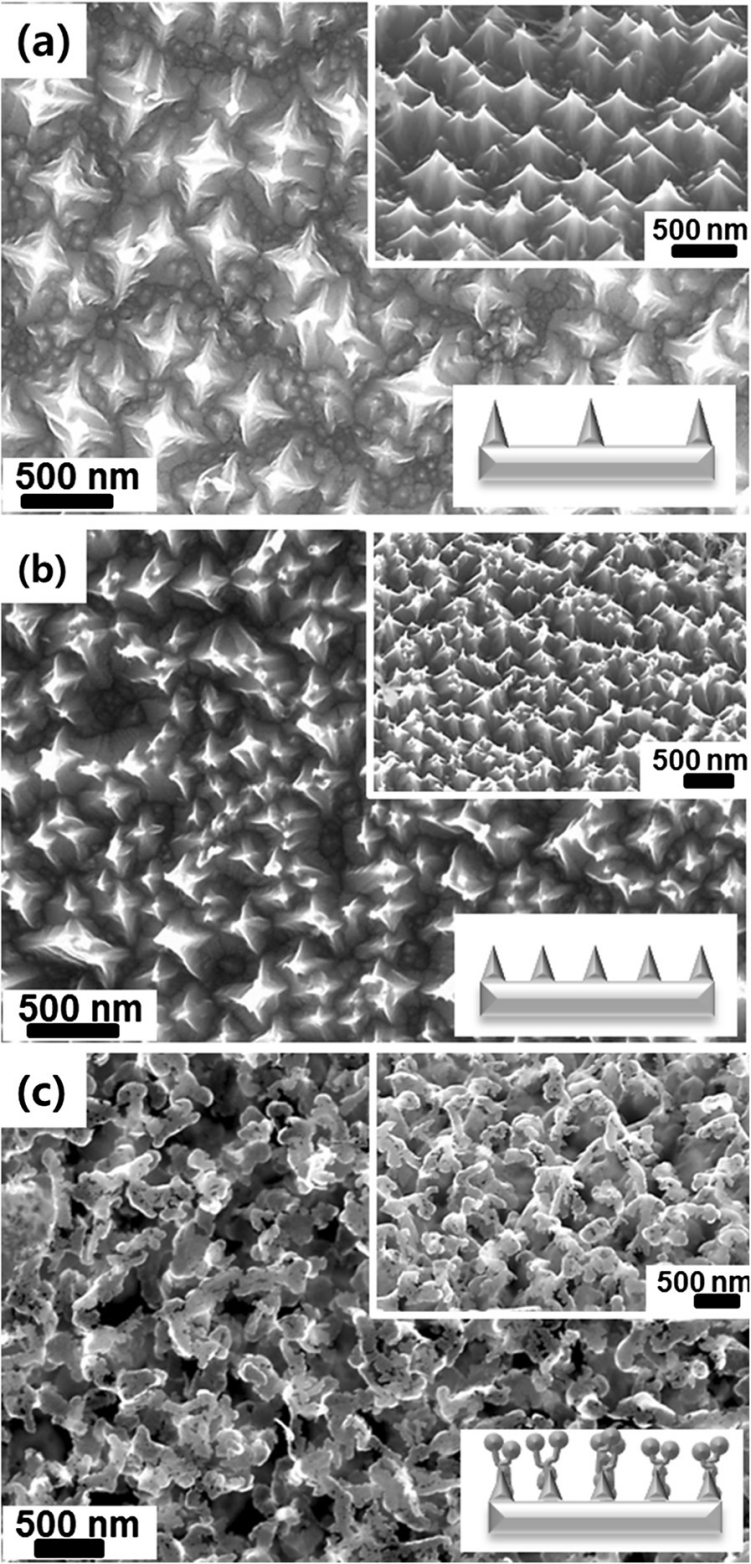
**FESEM images and schematics of the Si nanostructures.** Etching done at **(a)** 1,350°C, **(b)** 1,200°C, and **(c)** 1,100°C. Insets: tilted FESEM images and schematics of the Si nanostructures.

Both the flow rate of the hydrogen and argon mixture gas and the annealing temperature play important roles on the etching process [19]. To investigate the effects of gas flow rate on the Si etching degree, the hydrogen etching process was carried out at the various conditions of gas flow rate. Figure 2 shows the FESEM images of the fabricated Si nanostructures after the hydrogen etching processes at an annealing temperatures of 1,350°C. The flow rates to fabricate Si nanostructures were 0.5, 2.5, and 5.0 sccm (Figure 2a,b,c, respectively). The FESEM images exhibit that higher flow rate of mixture gas can induce stronger Si etching. As the flow rate is increased, non-regular Si nanostructures were formed: pyramid-like nanostructures were produced at 0.5 sccm (Figure 2a) and 2.5 sccm (Figure 2b), but aggregates of nanoparticles were fabricated on the surface at 5.0 sccm (Figure 2c). Based on this result, we fabricated Si nanostructures at a fixed flow rate of 0.5 sccm and different annealing temperatures of 1,350°C, 1,200°C, and 1,100°C. It can be seen that the fabricated Si nanostructures had aperiodic subwavelength structures with pyramid-like morphologies (Figure 3). At annealing temperatures from 1,200°C to 1,350°C, pyramid-shaped Si nanostructures were formed by hydrogen etching. The FESEM images and schematics demonstrate that the higher annealing temperature led to more perfect pyramid-shaped Si nanostructures and larger gaps between the Si nanopyramids (Figure 3a,b). However, no Si nanostructures were formed at the annealing temperature below 1,000°C, and bump-like Si nanostructures with additional nanoparticles on the apexes of the pyramids were produced at 1,100°C (Figure 3c). Due to the bump-like Si nanostructures, the total aspect ratio of the Si nanostructures was increased [4,5]. Moreover, the spacing between the Si nanostructures was decreased, which is beneficial to enhance the AR properties of the Si nanostructure [4,11].

Formation mechanism of the pyramid-shaped Si nanostructures can be explained as follows. An annealing of a Si plate under hydrogen environment weakens the bonds between Si atoms. As a result, SiH_*x*_ gases and radicals are formed easily through the reaction with hydrogen gas, leading to the etching of the Si plate [20]. During the hydrogen etching process, both etching and redeposition of the Si atoms/radicals occur and the Si surface was reproduced to have the most energetically stable shapes [18,21]. The (100) surface of Si is more rapidly etched than (110) and (111) surfaces [22]. As a result, pyramid-shaped Si nanostructures of which side faces comprise energetically stable (111) crystalline surfaces are formed [23]. However, non-perfect etching occurred at a relatively low annealing temperature of 1,100°C. Furthermore, SiH_*x*_ gases and radicals formed at such a low temperature can be redeposited on the Si nanostructure [18,24], leading to the formation of the bump-like structures on the apexes of the pyramid-like nanostructures as shown in Figure 3c.

**Figure 4:**
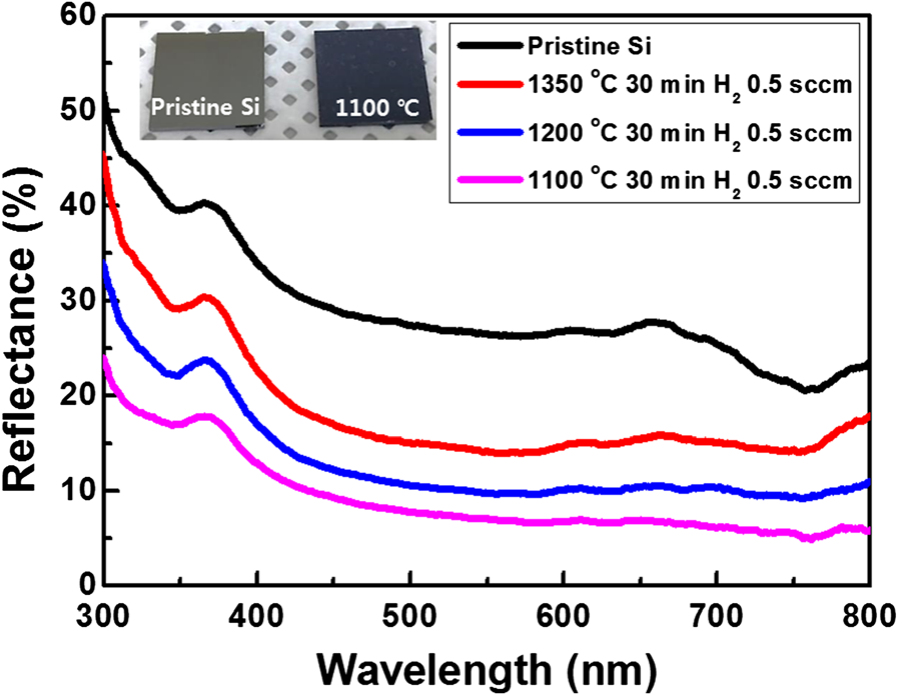
**Measured reflectance spectra of the fabricated Si nanostructures.** Inset: optical image of the pristine Si and Si nanostructure etched at 1,100°C.

The AR properties of the fabricated Si nanostructures were evaluated at normal incidences using a DR UV–Vis spectrometer. It is well known that pyramid, cone, and tip shapes with repeated two-dimensional subwavelength structures are the most effective to reduce the reflectance of sunlight at the interface between air and Si because they can change *n* smoothly [5,11,12]. The measured reflectance spectra of the fabricated Si nanostructures are displayed in Figure 4. Compared to pristine Si, the nanostructured surface significantly decreased the reflection in the UV–Vis region. In addition, the reflectance of the fabricated Si nanostructures was gradually reduced with the decrease in the annealing temperature, which is attributed to the fact that the spacing between the pyramid-like Si nanostructures was decreased when the annealing temperature was decreased [4,11]. The Si nanostructure etched at 1,100°C exhibited the best AR property: an average reflectance of approximately 6.8% was observed in the visible light region from 450 to 800 nm. Moreover, a pristine Si plate is shiny but the Si plate prepared at 1,100°C exhibited a dark blue color (inset of Figure 4).

**Figure 5:**
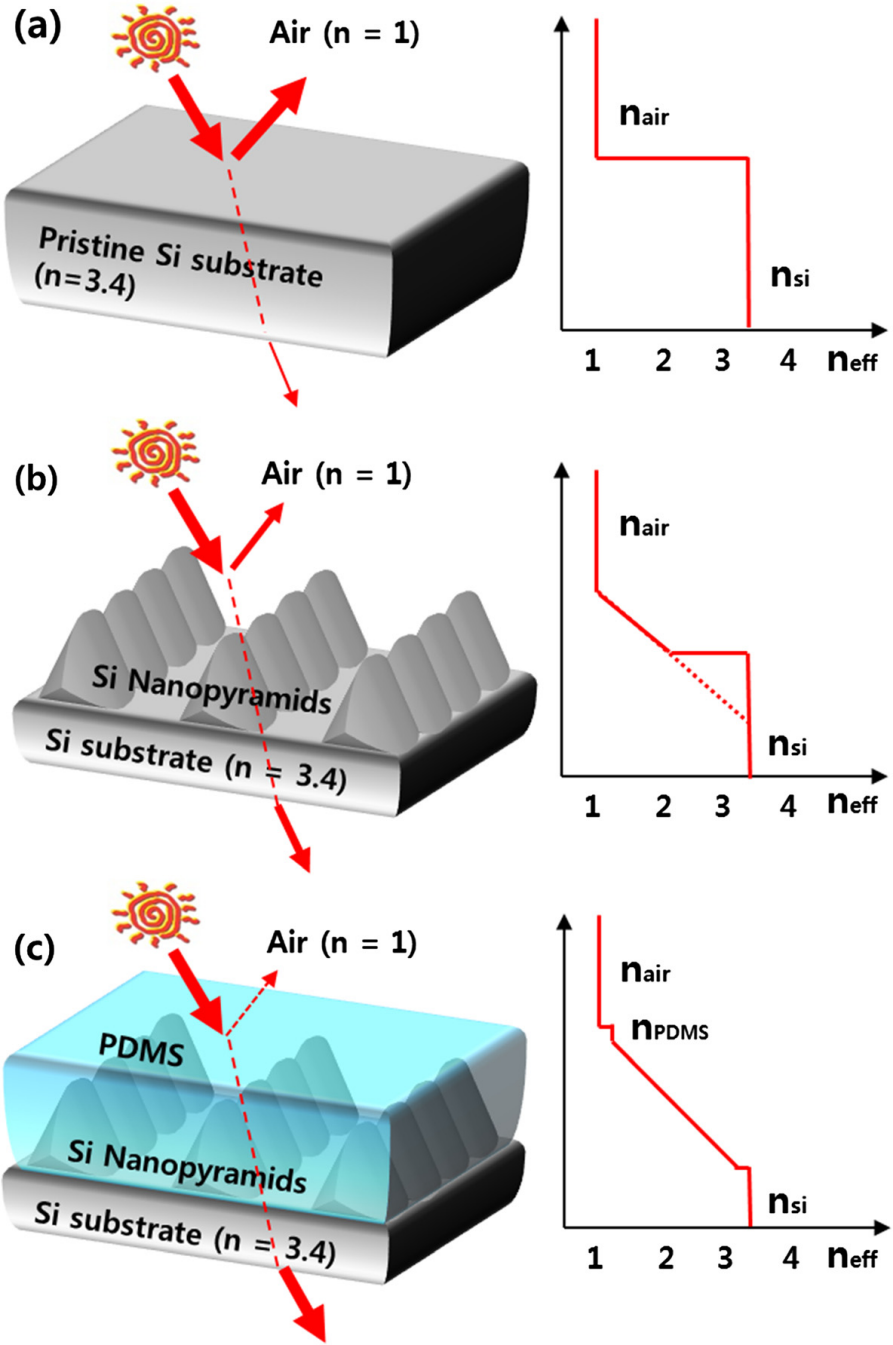
**Structure and effective refractive index profiles of various Si models. (a)** Pristine Si. **(b)** Si nanostructure. **(c)** Si nanostructure deposited via PDMS.

**Figure 6:**
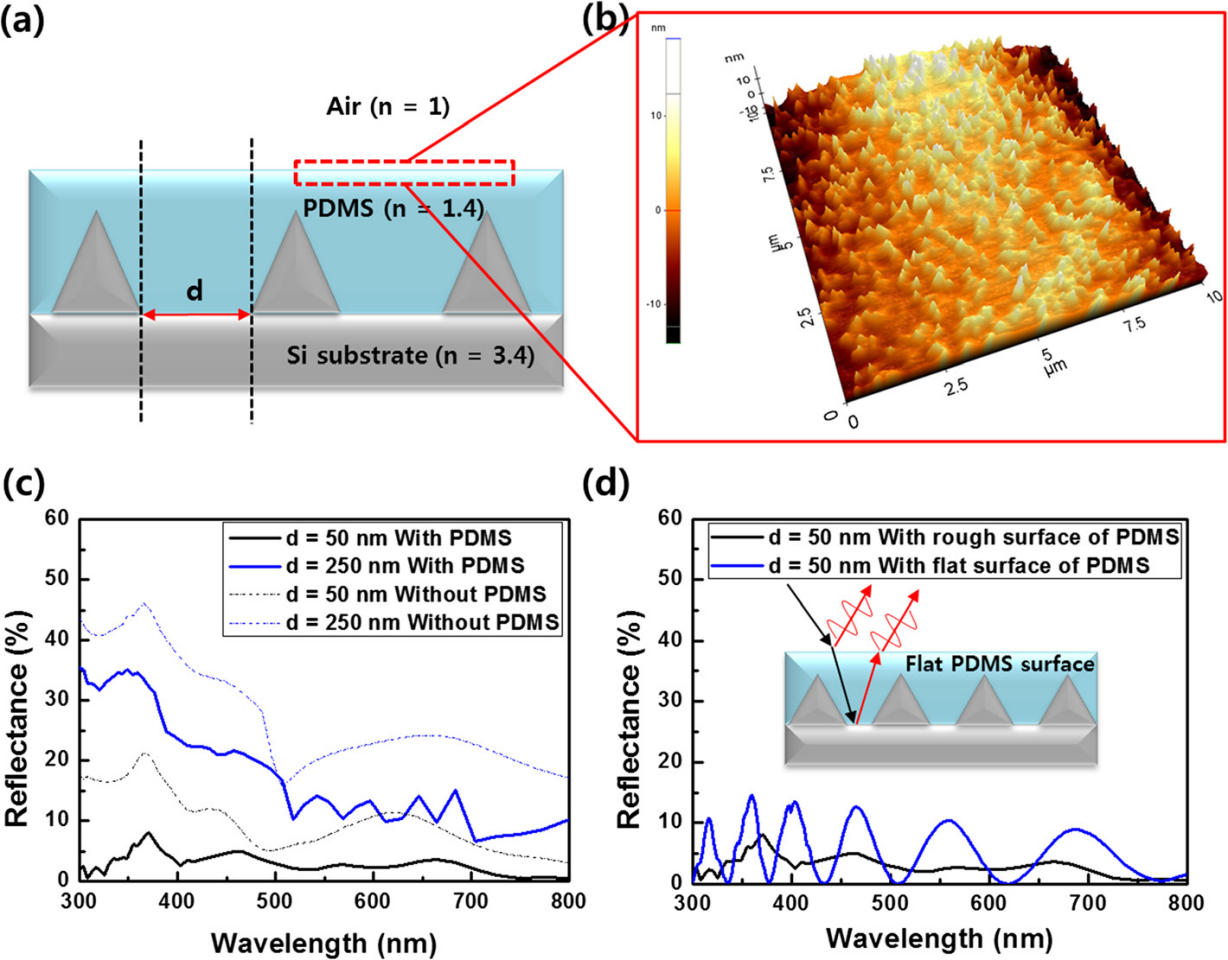
**Schematic of Si nanostructure, AFM image of the PDMS surface, and FDTD-simulated reflectance spectra. (a)** The schematic of buffer layer deposition on the non-compact nanopyramids array. **(b)** AFM image of the PDMS surface after the deposition on the Si nanostructures. The width and height of the Si nanopyramid are 300 and 250 nm in the simulation, respectively. FDTD-simulated reflectance spectra from the air-Si interface **(c)** before and after the PDMS deposition with increase in the distance between neighboring nanopyramids and **(d)** with rough and flat surfaces of PDMS. Inset: schematic of the flat PDMS surface on Si nanostructures.

**Figure 7:**
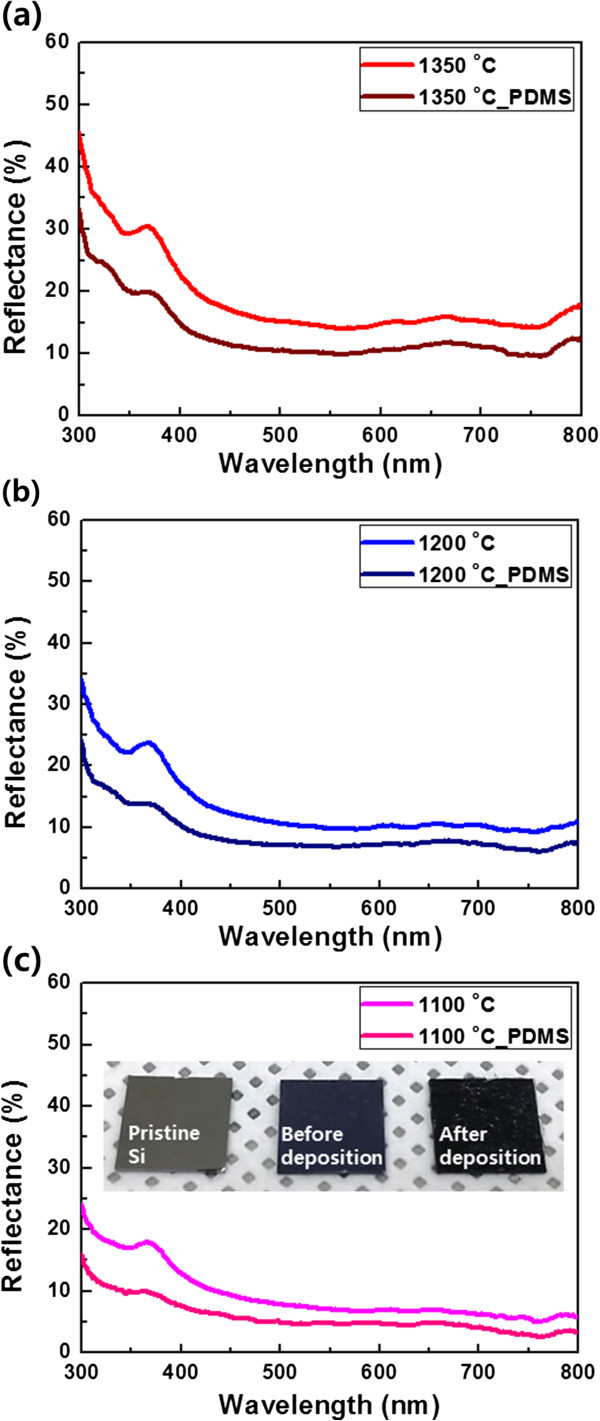
**Reflectance spectra before and after the PDMS deposition on the Si nanostructures.** Etching done at **(a)** 1,350°C, **(b)** 1,200°C, and **(c)** 1,100°C. Inset: optical image of the pristine Si and the Si nanostructures (etched at 1,100°C) before and after the PDMS deposition.

Figure 5 shows the effective refractive index (*n*_eff_) profiles of various Si structures. *n*_eff_ is defined by

(1) $$n_{\mathrm{eff}}=a\;n_{\mathrm{Si}}+b\;n_{\mathrm{air}},$$

where *a* and *b* are the area ratio of Si and air at a certain collinear position, and *n*_Si_ and *n*_air_ are the refractive index of the Si and air, respectively. For pristine Si, a relatively high reflectance is induced by the large difference in *n* at the air-Si interface between the two mediums. However, pyramid-like Si nanostructures lead to a smooth change of *n*_eff_ because the amount of air between the Si nanostructures is gradually decreased. Due to the smooth change of *n*_eff_ from air to the bottom of the Si nanostructures, light reflection is drastically decreased. Theoretically, a zero reflectance from the air-Si interface can be achieved if an ideal nanopyramid array is fabricated on a Si surface [25]. Such an ideal nanopyramid array results in a constantly varying *n* without a sharp change at the interface (dotted line in Figure 5b); however, achieving an ideal nanopyramid array is very difficult in reality. Particularly, nanopyramids are generally separated and some flat surface regions exist between the neighboring pyramids, as shown in Figure 6a. This non-compact nanopyramid structure prevents a smooth decline of *n*_eff_ at the air-Si interface, creating a discontinuity of *n*_eff_ (solid line in Figure 5b). The discontinuity of *n*_eff_ at the interface can be alleviated using a buffer layer between the air and Si nanostructures [26] (Figure 5c). If a buffer layer with *n* value between air and Si is deposited on the non-compact nanopyramids, the large difference in *n* between air and Si can be moderated by the buffer layer (Figure 5c). In our experiments, a Si-based polymer of PDMS was deposited on the fabricated Si nanostructures as a buffer layer because it has *n* of 1.4, which is an intermediate value between *n*_Si_ = 3.4 and *n*_air_ =1 [27]. After the PDMS layer deposition, the Si nanostructures (etched at 1,100°C) exhibited an average reflection of approximately 4.3% from 450 to 800 nm with a minimum reflectance of 2.5% at 760 nm (Figure 7c). This enhancement of the AR property could be clearly seen from the optical images of the Si substrates before and after the PDMS deposition. The dark blue color of the Si nanostructure before the deposition (center image of the inset in Figure 7c) transformed to a perfectly black color after the deposition (right image of the inset in Figure 7c). Consequently, the Si nanostructures coated with a PDMS buffer layer exhibited remarkably reduced reflectance at UV–Vis regions compared to a flat Si surface.

The AR properties of the non-compact nanopyramid structure and the effect of the buffer layer on the AR properties were analyzed with FDTD simulation. As shown in the simulation results (Figure 6c), the reflectance from the air-Si interface increases as the spacing (d in Figure 6a) between the neighboring nanopyramids increases. In addition, the FDTD simulation result also shows that a PDMS buffer layer further reduces the reflectance: the reflectance was reduced by approximately 5% over all the wavelength regions. These simulation results correspond well with the experimental results shown in Figure 7. In addition, although a buffer layer is deposited on the Si nanostructure, a reflection occurs at the surface of the buffer layer because of the difference in *n* between air and the PDMS buffer layer (see the small step in Figure 5c). However, we observed that surface of a PDMS layer was not perfectly flat. As shown in the AFM image (Figure 6b), the PDMS layer has a rough surface with the roughness of approximately 20 nm. This rough surface was naturally formed when the PDMS layer was coated on the Si nanostructures through the doctor blade technique. This rough surface of the PDMS layer induces a diffused reflection like the Si nanostructures on a Si plate and thus, the reflectance at the interface between air and PDMS layer is decreased [28]. The FDTD simulation result clearly demonstrates this fact (Figure 6d): relatively uniform low reflectance was obtained by the rough surface of the PDMS layer on the fabricated Si nanostructures (black line in Figure 6d). However, a flat surface of the PDMS layer with the thickness of 1 μm could induce the fluctuated and slightly high reflectance (blue line in Figure 6d) compared to that of the rough PDMS surface. These are because constructive and destructive interferences between reflections from the flat PDMS surface and the Si nanostructures are alternately occurred due to the flat surface of the PDMS layer (inset of Figure 6d). On the other hand, the rough surface of the PDMS layer could randomly scatter the reflections from the PDMS surface and the Si nanostructures, and thus, these arbitrarily scattered reflections by the rough PDMS surface could be dissipated through the destructive inference of themselves. Therefore, Si nanostructures and a PDMS buffer layer with a rough surface can dramatically improve the AR properties of a Si surface (Figure 7).

## Conclusions

Pyramid-shaped Si nanostructures were fabricated on a Si plate using a hydrogen etching process. Due to the nanopyramid structure, the Si surface exhibited a significantly low reflectance at UV and visible light regions. Furthermore, the discontinuity of *n*_eff_ at the air-Si interface could be reduced through the deposition of a Si-based polymer with a rough surface. Consequently, the AR properties of the Si nanostructures were further enhanced. The hydrogen etching method combined with a polymer coating can be easily scalable to a large surface and is a cheap process. Therefore, we believe that this method is useful for the practical applications to electro-optical devices that require low AR surfaces.

## Competing interests

The authors declare that they have no competing interests.

## Authors’ contributions

JMH carried out the design and fabrication of the experimental setups and drafted the manuscript. SHY assisted in the experiments. JHC and YHC carried out the simulation of the experimental setups using the finite difference time domain method. SOC supervised the whole study. All authors read and approved the final manuscript.
